# A randomized phase II study of weekly nab-paclitaxel plus gemcitabine or simplified LV5FU2 as first-line therapy in patients with metastatic pancreatic cancer: the AFUGEM GERCOR trial

**DOI:** 10.1186/s12885-015-1656-4

**Published:** 2015-10-06

**Authors:** Jean-Baptiste Bachet, Benoist Chibaudel, Franck Bonnetain, Pierre Validire, Pascal Hammel, Thierry André, Christophe Louvet, on behalf of the GERCOR group

**Affiliations:** 1 Paris-Sorbonne University, UPMC University Paris 06, Paris, France; 2 Department of hepatogastroenterology, Groupe hospitalier Pitié Salpêtrière, Paris, France; 3 Department of oncology, Hôpital Saint Antoine, Paris, France; 4 Head of methodology and quality of life in oncology department, Hôpital Universitaire de Besancon, EA 3181 Besancon, France; 5 Department of pathology, Institut Mutualiste Montsouris, Paris, France; 6 Department of digestive oncology, Hôpital Beaujon, Clichy, France; 7 Department of oncology, Institut Mutualiste Montsouris, Paris, France

**Keywords:** Pancreatic adenocarcinoma, Metastatic, Phase II, Nab-paclitaxel, sLV5FU2, hENT1

## Abstract

**Background:**

Metastatic pancreatic adenocarcinoma (PAC) prognosis remains dismal and gemcitabine monotherapy has been the standard treatment over the last decade. Currently, two first-line regimens are used in this setting: FOLFIRINOX and nab-paclitaxel plus gemcitabine. Increasing translational data on the predictive value of hENT1 for determining gemcitabine efficacy suggest that a non-gemcitabine-based regimen is favored in about 60 % of patients with PAC due to high resistance of PAC to this cytotoxic drug. This study aims to evaluate the efficacy of weekly nab-paclitaxel combined with gemcitabine or a simplified (s) LV5FU2 regimen in patients with previously untreated metastatic PAC.

**Methods/design:**

AFUGEM is a two-stage, open-label, randomized, multicenter, phase II trial. Patients with PAC who meet the inclusion criteria and provide written informed consent will be randomized in a 1:2 ratio to either nab-paclitaxel (125 mg/m^2^) plus gemcitabine (1000 mg/m^2^) given on days 1, 8, and 15 every 28 days or nab-paclitaxel (125 mg/m^2^) plus sLV5FU2 (leucovorin 400 mg/m^2^ followed by bolus 400 mg/m^2^ 5-fluorouracil and by 5-fluorouracil 2400 mg/m^2^ as an 46-h intravenous infusion) given on days 1 and 15 every 28 days. A total of 114 patients will be randomized to one of the treatment arms. The primary endpoint is progression-free survival at 4 months. Secondary outcomes are rate and duration of response, disease control, overall survival, safety, and quality of life. Potential biomarkers of gemcitabine (hENT1, dCK) and 5-fluorouracil (TS) efficacy will be assessed.

**Discussion:**

The AFUGEM trial is designed to provide valuable information regarding efficacy and tolerability of nab-paclitaxel plus gemcitabine and nab-paclitaxel plus sLV5FU2 regimens. Identification of potential predictive biomarkers of gemcitabine and 5-fluorouracil is likely to drive therapeutic decisions in patients with metastatic PAC.

**Trial registration:**

AFUGEM is registered at Clinicaltrials.gov: NCT01964534, October 15, 2013.

## Background

Pancreatic adenocarcinoma (PAC) is a fatal disease with poor prognosis and with the incidence increasing regularly in most of the western countries. PAC ranks as the fourth highest cause of death from cancer [1]. The 5 year survival is less than 5 % across all stages of disease [2, 3]. At diagnosis, 50–60 % of patients have distant metastases. In addition, up to 10 % of patients will develop metastases following a curative resection.

### Chemotherapy regimens in metastatic PAC

Monotherapy with gemcitabine has been the main therapeutic option over the last decade [4]. Several phase III studies have evaluated this regimen in combinations with multiple chemotherapy drugs and new targeted therapies. However, most of these studies were negative and failed to confer any added overall survival (OS) benefit in comparison to gemcitabine alone. Combinations of gemcitabine with fluoropyrimidine or derivative platinum have only been associated with a significant OS improvement in meta-analyses [5–7]. Conroy et al. showed that a gemcitabine-free triplet combination, FOLFIRINOX (5-fluorouracil [5FU], irinotecan, and oxaliplatin) achieve a significant progression-free survival (PFS) and OS benefit compared to gemcitabine alone in patients with metastatic PAC in a randomized phase II/III [8]. However, this trial included only patients with an Eastern Cooperative Oncology Group (ECOG) performance status (PS) of 0–1 and normal total bilirubin level [8].

Nab-paclitaxel is a solvent-free, albumin-bound 130-nm particle of paclitaxel. Preclinical studies in animals demonstrated lower toxicities for nab-paclitaxel, with the maximum-tolerated dose (MTD) approximately 50 % higher for nab-paclitaxel compared to paclitaxel [9]. At equitoxic doses, treatment with nab-paclitaxel achieved higher efficacy in an animal model as provided by paclitaxel dose.

The regimen of nab-paclitaxel plus gemcitabine showed promising antitumor activity and tolerable toxicity in patients with metastatic PAC in a phase II trial [10]. In the following MPACT randomized phase III study, evaluating the nab-paclitaxel plus gemcitabine vs gemcitabine monotherapy, median OS (8.5 months vs 6.7 months; *p* < 0.0001), median PFS (5.5 months vs 3.7 months; *p* < 0.0001) and the response rate (RR; 23 % vs 7 %; *p* < 0.0001) were significantly increased in the nab-paclitaxel plus gemcitabine arm [11]. The safety results were in concordance with those of the phase II [10]. The most common grade 3–4 adverse events in the combination arm and the gemcitabine arm were neutropenia (38 vs 27 %), fatigue (17 vs 7 %), and neuropathy (17 vs 1 %), respectively [11]. Nab-paclitaxel has subsequently been approved in the United States and the European Union for treatment of metastatic PAC.

### hENT1 − predictive biomarker of gemcitabine efficacy?

Understanding of the intracellular uptake and metabolism of gemcitabine led to further molecular investigation of these pathways for potential biomarkers affecting gemcitabine efficacy. Among biomarkers of potential clinical utility, the human equilibrative nucleoside transporter 1 (hENT1) has the most promising pre-clinical and clinical data suggesting its predictive value and thus value for guiding treatment decisions [12–15]. Gemcitabine is a hydrophilic prodrug. Its intracellular diffusion through the plasma membrane is low and requires specialized integral membrane nucleoside transporter proteins. Bi-directional hENTs play a key role in this processs and thus in the efficacy of gemcitabine [16]. Among these, hENT1 mediates the majority of gemcitabine transport [17]. Preclinical studies have suggested a positive correlation between hENT1 gene expression and gemcitabine chemosensitivity [17].

Data from phase II and phase III studies demonstrated no predictive value of hENT1 expression [18–20]. These findings put in question clinical utility of this biomarker. These studies of gemcitabine in metastatic and adjuvant setting used the SP120 rabbit monoclonal antibody [12–14], whereas three studies of adjuvant gemcitabine that showed an opposite benefit for hENT1 used the 10D7G2 monoclonal mouse antibody [18–20].

This difference is likely to be due to a lack of equivalency with a very poor rate of concordance between these two antibodies [21]. Moreover, a survival benefit for higher hENT1 expression with the 10D7G2 mouse antibody vs no benefit with a higher hENT1 expression with the SP120 rabbit antibody was reported [21].

More studies are necessary to evaluate the best method for assessing hENT1 expression and to develop a robust and reproducible scoring system for this biomarker. Despite different scoring systems used with the mouse antibody and its assessment restricted to adjuvant setting, up to 50–60 % of PACs are resistant to gemcitabine due to low or now expression of hENT1 [12–14]. Considering that the predictive value of hENT1 for gemcitabine efficacy is likely to be confirmed in the near future, the nab-paclitaxel plus gemcitabine regimen seems not to be the best treatment option for patients with low hENT1 expression.

### Rationale to develop the nab-paclitaxel plus sLV5FU2 regimen

For many years, 5FU has been considered as an efficient cytotoxic in PAC. After curative intent resection, gemcitabine-based and 5FU-based adjuvant chemotherapy showed similar survival advantage [22]. Moreover, fluoropyrimidines (capecitabine or 5FU) state key components of many first-line (e.g., FOLFIRINOX or gemcitabine plus capecitabine) and second-line regimens (e.g., OFF or 5FU plus MM-398) [7, 8, 23–26].

Nab-paclitaxel can be combined with fluoropyrimidine with tolerable toxicity. The combination of nab-paclitaxel plus capecitabine is currently being assessed in an adjuvant randomized phase II/III study in breast cancer patients at high risk of recurrence (ICE II/GBG 52 trial).

In randomized phase III trials, capecitabine had a better toxicity profile compared to 5FU bolus leading to a significantly lower incidence of grade 3–4 neutropenia and stomatitis but to an increased incidence of grade 3 hand-foot syndrome [27, 28].

To our knowledge, no randomized studies of capecitabine vs LV5FU2 have been reported. Yet, the LV5FU2 regimen was shown to have a better toxicity profile than 5FU bolus with less neutropenia, diarrhea, and stomatitis [29]. Therefore, the toxicity profile of the LV5FU2 regimen appears to be closer to the one of capecitabine than to that of 5FU bolus. In randomized phase III studies comparing XELOX to FOLFOX4 or FOLFOX6 in patients with metastatic colorectal cancer, XELOX was systematically associated with less grade 3–4 neutropenia but more grade 3–4 diarrhea and grade 3 hand-foot syndrome [30–32]. Based on these observations, the combination of LV5FU2 with nab-paclitaxel may present treatment option associated with more grade 3–4 neutropenia but probably less diarrhea and hand-foot syndrome.

In clinical practice, diarrhea is a common side effect in patients with PAC. In addition, peritoneal carcinomatosis is a common metastatic site in this setting that can limit oral administration of medication and increase the risk of digestive toxicity. The hand-foot syndrome is not easy to manage and requires frequently a dose-reduction of chemotherapy. Neutropenia represents the major dose-limiting toxicity, but the use of granulocyte colony stimulating factor (G-CSF) according to the EORTC recommendations may help in reducing the rate of chemotherapy-induced neutropenia and support the use of dose-intensity chemotherapy [33]. For these reasons, the LV5FU2 regimen in combination with nab-paclitaxel will probably be better tolerated than capecitabine.

### Translational study

In the past 10 years, an increasing number of translational studies on potential prognostic and/or predictive biomarkers in PAC have been published [34, 35]. As more treatment options are currently available in this setting, identification of robust biomarkers should be a priority. This may help in predicting treatment efficacy and/or in personalizing chemotherapy treatments. Several predictive biomarkers have been proposed for gemcitabine, 5FU, and nab-paclitaxel efficacy.

In addition to hENT1, deoxycitydine kinase (dCK) has been shown to represent a predictive biomarker of gemcitabine in patients with PAC. Interestingly, pooled hENT1 and dCK expression analysis provide supplementary predictive information as compared to separate analysis [13].

Numerous mechanisms are involved in the antitumor effect of 5FU. Among them, the most common is competitive inhibition of thymidylate synthase (TS). Predictive value of TS expression on 5FU sensitivity has been well described in vitro [36]. In vivo, a large meta-analysis in colorectal cancer has shown that a high level of TS is a worse predictive biomarker of OS in metastatic setting than in adjuvant setting [37]. Thus, TS expression could be assessed in pancreatic tumors using immunohistochemistry. To our knowledge, no studies have evaluated the predictive value of TS expression in metastatic PAC.

SPARC is a secreted protein acidic and rich in cysteine involved in cell matrix. A high expression of SPARC by peritumoral fibroblasts was described as a negative prognostic biomarker after curative surgery in patients with PAC [38]. On the contrary, over-expressed SPARC appeared to correlate with improved OS in patients treated with the gemcitabine plus nab-paclitaxel regimen in the original phase I/II trial of gemcitabine and nab-paclitaxel, and was suggested as a promising predictive biomarker of nab-paclitaxel efficacy [10]. The recently reported results from a randomized adjuvant study however have shown that a high SPARC expression is a negative predictive biomarker of adjuvant gemcitabine benefit [39]. Moreover, neither predictive nor prognostic value of SPARC expression level (in stromal fibroblasts tumoral cells or in plasma) was confirmed in the phase III MPACT trial [40].

The AFUGEM phase II trial is designed to assess the tolerability and efficacy of the sLV5FU2 plus nab-paclitaxel combination in comparison to the new standard regimen of gemcitabine plus nab-paclitaxel as first-line treatment in patients with metastatic PAC. Quality of life assessment and translational research is performed to determinate the best place and the possibilities of further clinical development of the nab-paclitaxel plus sLV5FU2 combination. Translational reserach will be exploratory and no statistical hypothesis plan was made given that the number of tumor samples available is still unknown.

## Methods/design

### Objectives

#### Primary objective

The primary objective is to assess PFS at 4 months in both treatment arms: nab-paclitaxel plus gemcitabine (arm A) and nab-paclitaxel plus simplified LV5FU2 (arm B). Survival is defined as the time interval between the randomization date and the date of either first documented disease progression (RECIST v1.1) or death of any cause, whatever occurs first [41]. Patients alive without progression will be censored at the last tumor assessment, either during study treatment period or during follow-up period.

### Secondary objective

The secondary objectives are to evaluate RR (RECIST v1.1), duration of RR, duration of disease control (DDC), OS, safety, health-related quality of life (HRQoL), and the prognostic and predictive value of SPARC, hENT1, dCK, and TS expression level in both treatment arms [41]. Survival is defined as the interval between the randomization date and the date of death from any cause. Patients still alive at the time of analysis will be censored at the last date known to be alive, either during study treatment period or during follow-up period. The grade of toxicity will be assessed using the NCI-CTC criteria v3.0. Quality of life will be studied by means of the EORTC QLQ C-30 questionnaire. A deterioration of scores for five-targeted dimensions: pain, physical and emotional functioning, fatigue, and appetite will be compared between the two treatment arms, while other dimensions will be regarded as exploratory. A 5-point deterioration in HRQoL scores will be considered as the minimal clinically important difference (MCID).

### Study design

The AFUGEM study is an open-label, randomized, multicenter phase II trial comparing weekly nab-paclitaxel with gemcitabine vs nab-paclitaxel with simplified LV5FU2 in patients with previously untreated metastatic PAC (Table 1 and Fig. 2).

**Table 1:** Detail of study regimens

Timing of administration	Day of administration	Drug dose
Arm A		
H0	1, 8, 15	nab-paclitaxel 125 mg/m^2^, 30 min IV infusion (maximum infusion time not exceeding 40 min)
H + 0.5	1, 8, 15	gemcitabine 1000 mg/m^2^ as a 30-min IV infusion
Arm B		
H0	1, 8, 15	nab-paclitaxel 125 mg/m^2^, 30 min IV infusion (maximum infusion time not exceeding 40 min)
H + 0.5	1, 15	folinic acid 400 mg/m^2^ (leucovorin, *l* + d racemic form, or *l* form 200 mg/m^2^) in 250 ml glucose 5 % solution, 2-h IV infusion
H + 2.5	1, 15	5FU bolus 400 mg/m^2^ in 100 ml glucose 5 % solution, 15 min IV infusion
H + 3	1–2, 15–16	5FU continuous infusion 2400 mg/m^2^, 46-h IV infusion

#### Enrolment

A total of 114 patients will be enrolled in a 1:2 ratio with 38 patients enrolled into the arm A and 76 patients enrolled into the arm B.

#### Stratification

Treatment assignment will be stratified, based on:

i. Center;
ii. ECOG PS 0–1 vs 2

### Eligibility criteria

#### Inclusion criteria

1. Signed and dated informed consent,
2. Patients willing and able to comply with protocol requirements,
3. Histologically or cytologically proven adenocarcinoma of the pancreas,
4. Stage IV disease,
5. No prior therapy for metastatic disease (in case of previous adjuvant therapy, interval between the end of chemotherapy and relapse must be >12 months),
6. At least one measurable or evaluable lesion as assessed by CT-scan or MRI according to RECIST v1.1,
7. Age ≥ 18 years,
8. ECOG PS 0 and 2,
9. Adequate hematologic function: neutrophils > 1.5 x 10^9^/L; platelets > 100 x 10^9^/L; hemoglobin ≥ 9 g/dL,
10. Adequate renal function: serum creatinine level < 150 μM,
11. Adequate liver function: AST (SGOT) and ALT (SGPT) ≤ 2.5 x ULN (≤5 x ULN in case of liver metastases), total bilirubin ≤ 1.5 x ULN, albumin ≥ 25 g/L,
12. Baseline evaluations performed before randomization: clinical and blood evaluations no more than 14 days prior to randomization, tumor assessment (CT-scan or MRI, evaluation of non-measurable lesions) no more than 21 days prior to randomization,
13. Female patients must be surgically sterile, or be postmenopausal, or must commit to using reliable and appropriate methods of contraception during the study and during at least 6 months after the end of study treatment (when applicable). All female patients with reproductive potential must have a negative pregnancy test (β HCG) within 72 h prior to starting nab-paclitaxel treatment. Breastfeeding is not allowed. Male patients must agree to use effective contraception in addition to having their partner use a contraceptive method as well during the trial and during at least 6 months after the end of the study treatment,
14. Registration with the French National Health Care System.

#### Exclusion criteria

1. Medical history or evidence of CNS metastasis upon physical examination, unless adequately treated (e.g., non-irradiated CNS metastasis, seizure not controlled with standard medical therapy),
2. Local or locally advanced disease (stage I to III),
3. Treatment with warfarin,
4. Uncontrolled hypercalcemia,
5. Pre-existing permanent neuropathy (NCI CTCAE grade ≥ 2),
6. Known dihydropyrimidine dehydrogenase deficiency,
7. Concomitant unplanned antitumor therapy (e.g., chemotherapy, molecular targeted therapy, immunotherapy),
8. Treatment with any other investigational medicinal product within 28 days prior to study entry,
9. Other serious and uncontrolled non-malignant disease (e.g., active infection requiring systemic therapy, coronary stenting or myocardial infarction, or stroke in the past 6 months),
10. HIV-infected patients or otherwise known to be HIV-positive with untreated hepatitis B or hepatitis C,
11. Medical history or active interstitial lung disease,
12. Other concomitant or previous malignancy, except: i/ adequately treated in-situ carcinoma of the uterine cervix, ii/ basal or squamous cell carcinoma of the skin, iii/ cancer in complete remission for > 5 years,
13. Patients with known allergy to any excipient of study drugs,
14. Concomitant administration of prophylactic phenytoin and live attenuated virus vaccine such as yellow fever vaccine.

#### Randomization

After having properly checked all eligibility criteria, stratification parameters, and having obtained patient written consent, patients will be randomized through an electronic case-report form (eCRF), using a minimization technique. The minimization algorithm takes into account the patients already randomized in order to allocate a subsequent treatment. A subgroup of patients who presents the same stratification variables that the patient to be randomized is isolated. The total number of patient in that subgroup is counted by stratification variables and by treatment group. The treatment group that is the less represented is selected by the system and attributed to the patient. The randomization result provided by the system is attributed in 80 % of the cases; otherwise the other treatment is attributed.

Randomized treatment will be confirmed by e-mail send to the investigator. All eligible patients must start study treatment within 7 days of randomization

### Ethics

This study is conducted in accordance to the standards of Good Clinical Practice (ICH-E6), the European Directive 2001/20/EC, the revised version of the Declaration of Helsinki, and local regulations. The protocol has been submitted and approved by the Agence Nationale de Sécurité du Médicament et des produits de santé (ANSM; French National Agency for Medicines and Health Product Safety) and the Comité de Protection des Personnes – Ile de France VI (French Ethics Committee).

Written informed consent is obtained from all patients prior to randomization.

### Treatment program

**Fig. 1:**
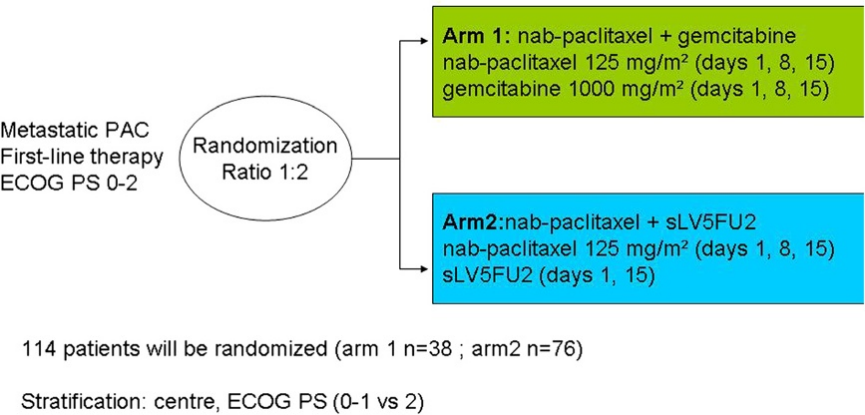
Design of study protocol

Patients will be treated on an outpatient basis with nab-paclitaxel plus gemcitabine (Arm A) or nab-paclitaxel plus sLV5FU2 (Arm B; Fig. 1).

### Arm A (Fig. 2; Table 1)

**Fig. 2:**
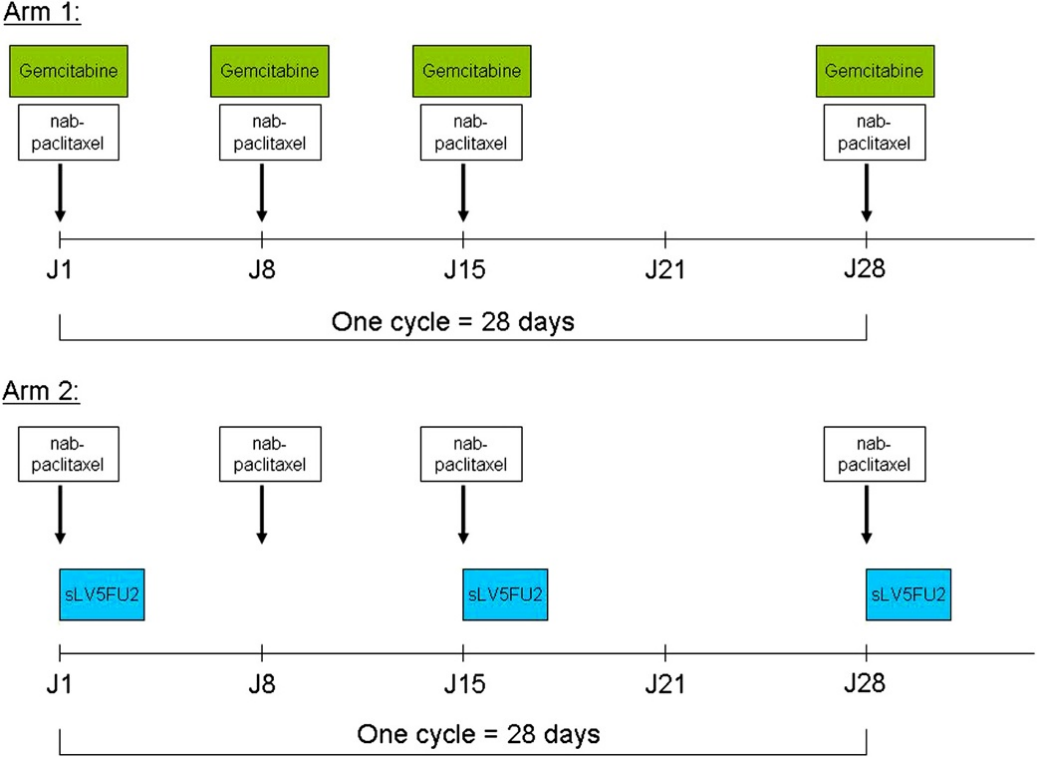
Design of study regimens

Arm A (nab-paclitaxel plus gemcitabine) consists of the following regimens: nab-paclitaxel at 125 mg/m^2^ plus gemcitabine at 1000 mg/m^2^ both administered as intravenous (IV) infusion over 30–40 min on days 1, 8, and 15, followed by 1 week of rest, every 28 days. Treatment continuation is intended until disease progression or limiting toxicity. If adverse events require dose interruption of:

- gemcitabine - nab-paclitaxel must be continued until progression;
- nab-paclitaxel - gemcitabine must be continued until progression;
- gemcitabine and nab-paclitaxel - a complete break in therapy is be allowed until disease progression.

### Arm B (Fig. 2; Table 1)

Arm B (nab-paclitaxel plus sLV5FU2) consists of the following regimens: nab-paclitaxel at 125 mg/m^2^ over 30–40 min IV infusion on days 1, 8, and 15, followed by 1 week of rest, every 28 days; folinic acid 400 mg/m^2^ (racemic form) or 200 mg/m^2^ (L-form) in 250 ml glucose 5 % solution given as a 2 h IV infusion on days 1 and 15; 5-FU bolus 400 mg/m^2^ in 100 ml glucose 5 % solution administered as a 15-min IV infusion on days 1 and 15; and 5-FU 2400 mg/m^2^ administered as continuous 46-h IV infusion on days 1–2 and 15–16. Treatment administration is intended until disease progression or limiting toxicity.

If adverse events require dose interruption of:

- sLV5FU2 - nab-paclitaxel must be continued until progression;
- nab-paclitaxel - sLV5FU2 must be continued until progression;
- sLV5FU2 and nab-paclitaxel - a complete break in therapy is allowed until disease progression.

In both arms, doses of the first cycle will be adapted according to ECOG PS at inclusion:

- Patients with ECOG PS 0 or 1 will receive a full dose at the first cycle,
- Patients with ECOG PS 2 will receive reduced dose by 20 % at the first cycle (nab-paclitaxel 100 mg/m^2^),
- In absence of toxicity grade 2–4 during the first cycle, patients with ECOG PS 2 will receive a full dose at the second cycle.

A systematic G-CSF prophylaxis is recommended in both arms according to EORTC recommendations. The type of G-CSF and treatment duration will be determined according to local standard of care in each center.

#### Assessment of tumor response

Tumor response measured using chest-abdominal CT-scan (or MRI) using RECIST v1.1. For each patient, the same method of assessment and the same technique must be used to evaluate each lesion throughout the entire treatment period. If more than one method is used, the most accurate method according to RECIST v1.1 will be selected when recording data. Baseline total tumor burden must be assessed no more than 21 days before randomization and no more than 28 days before starting study treatment.

The following items must be available:

*At baseline and 14 days follow-up*:

- Weight, blood pressure, ECOG PS, neurological examination, hematology, coagulation, and tumor markers (CA 19–9 and CEA), radiologic characteristics (assessed by CT, MRI according to RECIST v1.1), EORTC QLQ C-30 questionnaire,
- Serum pregnancy test in women of childbearing potential performed within 72 h prior to nab-paclitaxel treatment,
- Inclusion and exclusion criteria,
- Paraffin embedded tumor tissue in eligible patients.

*During study treatment:*

*Prior to the schedule dosing (<48 h):*

- Concomitant treatment information,
- Weight, blood pressure, ECOG PS, complete and differential blood counts, hematology, serum creatinine, total and indirect bilirubin, AST, ALT, ALP, EORTC QLQ C-30 questionnaire (at day 1 of every cycle), and toxicity.

*Every 2 months:*

- Weight, blood pressure, ECOG PS radiological tumor assessment, complete and differential blood counts, hematology, albumin plasma, CA 19–9, and CEA levels, toxicity, and concomitant medication.

If there is a suspicion of disease progression based on clinical or laboratory findings before the next scheduled assessment, an unscheduled assessment should be performed. All tumor assessments after baseline will be done within +/−7 days of the scheduled visit. If the patient inadvertently misses a prescribed tumor evaluation or a technical error prevents the evaluation, this patient may continue treatment until the next assessment and an unscheduled assessment should be planned as soon as possible.

*End of treatment (28 days after the last dose of any study drug with a +/− 3 day window):*

- Date and reason for end of the study treatment,
- Weight, blood pressure, ECOG PS (Appendix 17.3), complete and differential blood counts, hematology, albumin plasma level, EORTC QLQ C-30 questionnaire, toxicity, and concomitant medication.

*Follow-up*:

- Date of disease progression (if patient is withdrawn for reasons other than progression),
- Date of initiation and type of any second and subsequent lines of therapy,
- Date of death.

### Statistical analysis

The primary analysis will be performed on modified intention-to-treat (mITT) population, i.e., all randomized patients regardless of their eligibility and received treatment. Confirmative analyses will be conducted firstly in the ITT population (patients not assessable and these who dropped out before month 4 will be considered as progressive) and in the per protocol (PP) population defined as patients who have received at least one dose of allocated treatment and have no major deviations from the protocol. Analyses of tolerance will be conducted in all patients who have received at least one dose of allocated treatment. Unless otherwise indicated all analyses will present data by treatment arm.

QoL analyses will be conducted in the mITT2 population defined as all randomized patients whatever eligibility criteria were fulfilled and study treatment received with at least one QoL questionnaire completed at baseline. The mITT population will be used for the analyses of all efficacy endpoints.

The following evaluable patient populations are defined for selected endpoints:

- Tumor response will be evaluated in randomized patients with measurable disease at baseline,
- CA 19–9 response will be evaluated in randomized patients with available CA 19–9 assessment at baseline.

Prior to locking the database, a data review meeting will be planned in order to review individual data and validate the Statistical Analysis Plan (SAP). All the deviations from protocol definitions (if any) will be listed and defined as major or minor deviations in the SAP.

With regard to the safety evaluation, the analysis will be performed in the total treated set in order to document the safety when the treatment is actually received. Total treated set is defined as all patients who received at least one administration of assigned treatment. The safety population will be used for reporting of safety data and treatment exposure data. Selected efficacy analyses will be repeated for the ITT population, PP population, and for subgroups.

Statistical analyses will be performed using eCRF data collected until a clinical cut-off date that is defined when the number of events required for the interim and final analysis of the efficacy variables will be achieved.

Continuous variables will be summarized using descriptive statistics, i.e., number of patients with available data (N), mean, median, standard deviation (S.D.), 25–75 % quartile (Q1-Q3), minimum, and maximum. Continuous variables could be transformed to categorical variables using the median or using conventional cut-offs from bibliography or clinical practice. Frequencies in tables will be presented by arm, total frequency, percentages, and missing modality. Qualitative variables will be summarized by means of counts and percentages. Unless otherwise stated the calculation of proportions will based on the sample size of the population of interest. 95 % confidence interval (CI) will be calculated for the observed 4-month PFS.

Kaplan Meier curves will be used to describe event-free rates over time. Median event-free times by treatment arm will be reported with 95 % CI, if the number of events allows the estimation of the median. The confidence interval for the median survival time will be calculated according to Brookmeyer, R. and Crowley, J. (1982). Event rates at specified time points will be estimated from the Kaplan-Meier curve and will be reported with 95 %. The standard error will be estimated by the Greenwood formula and the log-log transformation will be used to compute confidence intervals. As exploratory purpose only, univariate Cox analyses will be done to compute hazard ratio and its 95 % CI. Follow-up will be estimated using the reverse Kaplan-Meier method, and will be described using the median with its 95 % CI.

Clinical and demographic data will be described using rules form. The statistical parameters mean, median, SD, and interquartile range and range will be presented for continuous baseline variables. For categorical baseline variables, corresponding frequencies (n, %) will be calculated. All baseline variables listed below will be summarized by treatment arm.

The dose-intensity (DI) of a drug is calculated based on the number of cycles actually received by the patient. The relative DI is calculated as the ratio of the DI to the DI indicated in the protocol. The DI indicated in the protocol is obtained as the dose specified per cycle (mg/m^2^).

#### Safety

The following adverse events related to treatment will be reported:

(i) Any adverse events,
(ii) Any serious adverse events,
(iii) Any serious adverse events related to study treatment,
(iv) Any NCI-CTC grade 3 or 4 adverse events,
(v) Any adverse events causing discontinuation of study treatment,
(vi) Any adverse events causing a dose reduction of study medication,
(vii) Any adverse events leading to death.

### Sample size

According to Fleming 2-stage design with a one-sided 5 % type I error and power of 80 %, 72 patients in arm B (nab-paclitaxel plus sLV5FU2) will need to be randomized in order to test the following hypotheses:

- H0 (null): a PFS rate at 4 months of 35 % (uninteresting to pursue any further investigation),
- H1 (alternative): a PFS rate at 4 months of 50 % (warrants further investigation in a phase III trial).

The hypotheses regarding an anticipated PFS rate at 4 months of 50 % and an uninteresting rate of 35 % are based on the observed PFS in metastatic PAC with ECOG PS 0–2 and treated with first-line gemcitabine [39, 42].

The control arm will serve as calibration that the populations in the two arms are similar: no statistical comparison is planned between the two arms.

### Stage 1

In arm B, after 4-month follow-up of the first 15 recruited patients:

- if only one patient who is alive is free of progression at 4 months (6.7 %), the treatment would be declared uninteresting. No more additional patient will be included in this arm and the study will be stopped. Standard treatment (at the investigator’s discretion) should be given to the potential progression-free patient.
- if up to 12 patients are alive and free of progression at 4 months (80 %), 57 additional patients will be randomized to arm B;
- if 13 or more patients are alive and progression free at 4 months (86.7 %), the treatment would be declared a success and deemed worthy of further phase III study, however an additional 57 patients will be allocated to arm B.

The probability to conclude for efficacy at the end of stage 1 is α1 = 0 %, whereas *p* = 35.0 %.

An early interim analysis is planned for early determination of the efficacy and safety data.

### Stage 2

In arm B, after 4-month follow-up of 72 randomized patients:

- if 32 or less patients are progression free at 4 months (≤44.4 %), the treatment would be declared uninteresting,
- if 33 or more patients are progression free at 4 months (≥45.8 %), the treatment would be regarded as interesting for further evaluation in a phase III trial.

The probability to conclude for inefficacy at the end of stage 1 is β2 = 20.4 %, whereas *p* = 50.0 %. The probability to conclude for efficacy at the end of stage 1 is α2 = 3.7 %, whereas *p* = 35.0 %.

With an expected 5 % drop out/ non-evaluable rate at 4 months, a total of 114 patients are required (arm A: *N* = 38; arm B: *N* = 76).

Final and specific statistical plan dedicated to QoL analyses will be written before data frozen.

An analysis will be realized to determine the mechanism of missing data

The method of scoring could take into account the mechanism of missing data highlighted for example using multiple imputations taking into account factors linked to the occurrence of missing data (as a sensitivity analysis).

A descriptive analysis of the scores obtained for each questionnaire at baseline and at each follow-up will be performed using number (N), mean (SD), and median (range) for all patients and according to the treatment arm.

The longitudinal analysis of HRQoL will be performed according to the time to HRQoL score deterioration (TUDD) approach. The TUDD will be defined as the time from inclusion in the study to the first deterioration of a least 5-point MCID of the HRQoL score as compared to the baseline score, with no further improvement as compared to the baseline score.

In sensitivity HRQoL analysis, TUDD defined as HRQoL deterioration-free survival including all-cause death as an event, will be explored.

The TUDD will be estimate according to the Kaplan-Meier estimation method. As exploratory purpose only, univariate Cox analyses will be done to compute hazard ratio and its 95 % CI.

As an exploratory univariate and multivariate Cox regression models will be performed in order to estimate hazard ratio with 95 % CI investigate potential factors independently associated with the TUDD including time to progression and time to the first grade 3–4 toxicities.

#### Translational research project

Paraffin-embedded tumor tissue will be collected prior to treatment and stored centrally in the tumor bank at Institute Mutualiste Montsouris, Paris. A systematic translational research with analysis of immunohistochemistry (blinded with respect to treatment and patient response) with regard to hENT1, dCK, and TS expression will be performed using previously reported methods [13, 37]. Analysis of SPARC expression will be performed if feasible.

## Discussion

After more than 10 years of failure to improve the efficacy of chemotherapy regimens in patients with advanced PAC, FOLFIRINOX and nab-paclitaxel plus gemcitabine have emerged as two new treatment standard based on the results of two phase III trials [8, 11]. However, the indications of FOLFIRINOX are limited to patients with good PS (ECOG 0–1) and normal bilirubin level [8]. Given that most patients with metastatic PAC have a poor PS at diagnosis, FOLFIRINOX is therefore an option only for a minority of patients. In the MPACT phase III study, patient selection criteria were less restrictive and these with a Karnofsky PS score of 70 or more were considered eligible [11]. Moreover, predefined sub-groups analysis data suggested that nab-paclitaxel treatment effect favored patients with negative prognostic factors (Karnofsky PS, liver metastases, number of metastatic sites, and level of CA 19–9). The recent results of the CONKO-003 and NAPOLI-1 trials demonstrated the interest of investigating these regimen combinations in the second-line setting [25, 26]. Currently, gemcitabine, fluoropyrimidine, irinotecan, oxaliplatin, and nab-paclitaxel are the five available cytotoxic drugs for treatment of patients with metastatic PAC that showed anti-tumor efficiency.

Different therapeutic options can be considered to offer further improvements in the results of PAC treatment. One option will be to increase the number of drugs used or to optimize the therapeutic sequence. Such strategies have been evaluated in a phase I study combining 5FU, oxaliplatin and nab-paclitaxel [43], and in a phase II trial with sequential administration of gemcitabine alone followed by FOLFIRI3 regimen during a 2 months alternative period [44]. The promising results reported in those studies led to the development of numerous trials: e.g., NABPLAGEM (NCT01893801; combination of nab-paclitaxel, cisplatin, and gemcitabine), GABRINOX (NCT01964287; alternative administration of nab-paclitaxel plus gemcitabine and FOLFIRINOX), and FIRGEMAX (alternative administration of nab-paclitaxel plus gemcitabine and FOLFIRI3). A major concern of these strategies will be probably the profile of tolerance that will limit the administration of these treatments to patients with 0–1 ECOG PS. It seems necessary therefore to develop more easily tolerable combinations for older patients or those with ECOG PS ≥ 2.

Another option will be to optimize the use of the available drugs using predictive biomarkers. Such strategy of selecting “the right treatment for the right patient” will allow to increase the efficacy and to limit the toxicity. It is of particular interest in metastatic PAC as most of patients have symptoms and are in PS ≥ 2 at diagnosis. The AFUGEM trial was designed to optimize the nab-paclitaxel combination according to predictive biomarkers. Recent disappointing results on the hENT1 predictive value with the SP120 antibody in patients with PAC have raised many questions and should be interpreted with caution [18–21].

## Conclusion

The AFUGEM trial is designed for patients with metastatic PAC. Two strategies are compared: nab-paclitaxel plus gemcitabine and nab-paclitaxel plus sLV5FU2 in order to provide important information on the safety and efficacy of the nab-paclitaxel plus sLV5FU2 combination. Parallel translational research will assess the predictive value of biomarkers of gemcitabine (hENT1, dCK) and 5FU (TS) efficacy to better determine the best drug to be added to nab-paclitaxel in individual patients.

## Abbreviations

PAC: Pancreatic adenocarcinoma
nab-paclitaxel: Nanoparticle albumin-bound paclitaxel
FOLFIRINOX: FOLinic acid-Fluorouracil-IRINotecan-OXaliplatin
LV5FU2: Leucovorin, 5-fluorouracil
PFS: Progression-free survival
MTD: Maximum tolerated dose
RECIST: Response evaluation criteria in solid tumors
hENT-1: Human equilibrative nucleoside transporter 1
dCK: Deoxycitydine kinase
TS: Thymidilate synthase
SPARC: Secreted protein acidic and rich in cysteine

## References

[1] Bouvier AM, Remontet L, Jougla E, Launoy G, Grosclaude P, Buémi A, Tretarre B, Velten M, Dancourt V, Menegoz F, Guizard AV, Macé Lesec’h J, Peng J, Bercelli P, Arveux P, Estève J, Faivre J (2004). Incidence of Gastrointestinal Cancers in France. Gastroenterol Clin Biol.

[2] Coleman MP, Gatta G, Verdecchia A, Estève J, Sant M, Storm H, Allemani C, Ciccolallo L, Santaquilani M, Berrino F, EUROCARE Working Group: EUROCARE Working Group (2003). EUROCARE-3 Summary: Cancer Survival in Europe at the End of the 20th Century. Ann Oncol.

[3] Carpelan-Holmström M, Nordling S, Pukkala E, Sankila R, Lüttges J, Klöppel G, Haglund C (2005). Does Anyone Survive Pancreatic Ductal Adenocarcinoma? A Nationwide Study re-Evaluating the Data of the Finnish Cancer Registry. Gut.

[4] Burris HA, Moore MJ, Andersen J, Green MR, Rothenberg ML, Modiano MR, Cripps MC, Portenoy RK, Storniolo AM, Tarassoff P, Nelson R, Dorr FA, Stephens CD, Von Hoff DD (1997). Improvements in Survival and Clinical Benefit with Gemcitabine as First-Line Therapy for Patients with Advanced Pancreas Cancer: a Randomized Trial. J Clin Oncol.

[5] Heinemann V, Boeck S, Hinke A, Labianca R, Louvet C (2008). Meta-analysis of randomized trials: evaluation of benefit from gemcitabine-based combination chemotherapy applied in advanced pancreatic cancer. BMC Cancer.

[6] Banu E, Banu A, Fodor A, Landi B, Rougier P, Chatellier G, Andrieu JM, Oudard S (2007). Meta-Analysis of Randomised Trials Comparing Gemcitabine-Based Doublets versus Gemcitabine alone in Patients with Advanced and Metastatic Pancreatic Cancer. Drugs Aging.

[7] Cunningham D, Chau I, Stocken DD, Valle JW, Smith D, Steward W, Harper PG, Dunn J, Tudur-Smith C, West J, Falk S, Crellin A, Adab F, Thompson J, Leonard P, Ostrowski J, Eatock M, Scheithauer W, Herrmann R, Neoptolemos JP (2009). Phase III Randomized Comparison of Gemcitabine versus Gemcitabine plus Capecitabine in Patients with Advanced Pancreatic Cancer. J Clin Oncol.

[8] Conroy T, Desseigne F, Ychou M, Bouché O, Guimbaud R, Bécouarn Y, Adenis A, Raoul JL, Gourgou-Bourgade S, de la Fouchardière C, Bennouna J, Bachet JB, Khemissa-Akouz F, Péré-Vergé D, Delbaldo C, Assenat E, Chauffert B, Michel P, Montoto-Grillot C, Ducreux M, Groupe Tumeurs Digestives of Unicancer; PRODIGE Intergroup (2011). FOLFIRINOX versus gemcitabine for metastatic pancreatic cancer. N Engl J Med.

[9] Desai N, Trieu V, Yao Z, Louie L, Ci S, Yang A, Tao C, De T, Beals B, Dykes D, Noker P, Yao R, Labao E, Hawkins M, Soon-Shiong P (2006). Increased antitumor activity, intratumor paclitaxel concentrations, and endothelial cell transport of cremophor-free, albumin-bound paclitaxel, ABI-007, compared with cremophor-based paclitaxel. Clin Cancer Res.

[10] Von Hoff DD, Ramanathan RK, Borad MJ, Laheru DA, Smith LS, Wood TE, Korn RL, Desai N, Trieu V, Iglesias JL, Zhang H, Soon-Shiong P, Shi T, Rajeshkumar NV, Maitra A, Hidalgo M (2011). Gemcitabine plus nab-paclitaxel is an active regime, in patients with advanced pancreatic cancer: a phase I/II trial. J Clin Oncol.

[11] Von Hoff DD, Ervin T, Arena FP, Chiorean EG, Infante J, Moore M, Seay T, Tjulandin SA, Ma WW, Saleh MN, Harris M, Reni M, Dowden S, Laheru D, Bahary N, Ramanathan RK, Tabernero J, Hidalgo M, Goldstein D, Van Cutsem E, Wei X, Iglesias J, Renschler MF (2013). Increased survival in pancreatic cancer with nab-paclitaxel plus gemcitabine. N Engl J Med.

[12] Farrell JJ, Elsaleh H, Garcia M, Lai R, Ammar A, Regine WF, Abrams R, Benson AB, Macdonald J, Cass CE, Dicker AP, Mackey JR (2009). Human equilibrative nucleoside transporter 1 levels predict response to gemcitabine in patients with pancreatic cancer. Gastroenterology.

[13] Maréchal R, Bachet JB, Mackey JR, Dalban C, Demetter P, Graham K, Couvelard A, Svrcek M, Bardier-Dupas A, Hammel P, Sauvanet A, Louvet C, Paye F, Rougier P, Penna C, André T, Dumontet C, Cass CE, Jordheim LP, Matera EL, Closset J, Salmon I, Devière J, Emile JF, Van Laethem JL (2012). Levels of gemcitabine transport and metabolism proteins predict survival times of patients treated with gemcitabine for pancreatic adenocarcinoma. Gastroenterology.

[14] Greenhalf W, Ghaneh P, Neoptolemos JP, Palmer DH, Cox TF, Lamb RF, Garner E, Campbell F, Mackey JR, Costello E, Moore MJ, Valle JW, McDonald AC, Carter R, Tebbutt NC, Goldstein D, Shannon J, Dervenis C, Glimelius B, Deakin M, Charnley RM, Lacaine F, Scarfe AG, Middleton MR, Anthoney A, Halloran CM, Mayerle J, Oláh A, Jackson R, Rawcliffe CL, Scarpa A, Bassi C, Büchler MW, European Study Group for Pancreatic Cancer (2014). Pancreatic cancer hENT1 expression and survival from gemcitabine in patients from the ESPAC-3 trial. J Natl Cancer Inst.

[15] Wei CH, Gorgan TR, Elashoff DA, Hines OJ, Farrell JJ, Donahue TR (2013). A meta-analysis of gemcitabine biomarkers in patients with pancreaticobiliary cancers. Pancreas.

[16] Barnes K, Dobrzynski H, Foppolo S, Beal PR, Ismat F, Scullion ER, Sun L, Tellez J, Ritzel MW, Claycomb WC, Cass CE, Young JD, Billeter-Clark R, Boyett MR, Baldwin SA (2006). Distribution and functional characterization of equilibrative nucleoside transporter-4, a novel cardiac adenosine transporter activated at acidic pH. Circ Res.

[17] Mackey JR, Mani RS, Selner M, Mowles D, Young JD, Belt JA, Crawford CR, Cass CE (1998). Functional nucleoside transporters are required for gemcitabine influx and manifestation of toxicity in cancer cell lines. Cancer Res.

[18] Poplin E, Wasan H, Rolfe L, Raponi M, Ikdahl T, Bondarenko I, Davidenko I, Bondar V, Garin A, Boeck S, Ormanns S, Heinemann V, Bassi C, Evans TR, Andersson R, Hahn H, Picozzi V, Dicker A, Mann E, Voong C, Kaur P, Isaacson J, Allen A (2013). Randomized, multicenter, phase II study of CO-101 versus gemcitabine in patients with metastatic pancreatic ductal adenocarcinoma: including a prospective evaluation of the role of hENT1 in gemcitabine or CO-101 sensitivity. J Clin Oncol.

[19] Ormanns S, Heinemann V, Raponi M, Isaacson J, Laubender RP, Haas M, Kruger S, Kleespies A, Mann E, Bartosiewicz M, Kirchner T, Boeck S (2014). Human equilibrative nucleoside transporter 1 is not predictive for gemcitabine efficacy in advanced pancreatic cancer: translational results from the AIO-PK0104 phase III study with the clone SP120 rabbit antibody. Eur J Cancer.

[20] Sinn M, Sinn BV, Stieler J, Pelzer U, Striefler JK, Oettle H, Bahra M, Dörken B, Denkert C, Blaeker H, Riess H, Lohneis P (2014). Hent1 expression in patients with pancreatic cancer treated with gemcitabine after curative intended resection: Results from the CONKO-001 trial. J Clin Oncol.

[21] Svrcek M, Cros J, Maréchal R, Bachet JB, Fléjou JF, Demetter P: hENT1 testing in pancreatic ductal adenocarcinoma: a comparison between the murine and the rabbit antibodies. Histopathology 2014, in press. 10.1111/his.12577 25298108

[22] Neoptolemos JP, Stocken DD, Bassi C, Ghaneh P, Cunningham D, Goldstein D, Padbury R, Moore MJ, Gallinger S, Mariette C, Wente MN, Izbicki JR, Friess H, Lerch MM, Dervenis C, Oláh A, Butturini G, Doi R, Lind PA, Smith D, Valle JW, Palmer DH, Buckels JA, Thompson J, McKay CJ, Rawcliffe CL, Büchler MW, European Study Group for Pancreatic Cancer (2010). Adjuvant chemotherapy with fluorouracil plus folinic acid vs gemcitabine following pancreatic cancer resection: a randomized controlled trial. JAMA.

[23] Taïeb J, Lecomte T, Aparicio T, Asnacios A, Mansourbakht T, Artru P, Fallik D, Spano JP, Landi B, Lledo G, Desrame J (2007). FOLFIRI.3, a new regimen combining 5-fluorouracil, folinic acid and irinotecan, for advanced pancreatic cancer: results of an Association des Gastro-Enterologues Oncologues (Gastroenterologist Oncologist Association) multicenter phase II study. Ann Oncol.

[24] Dahan L, Bonnetain F, Ychou M, Mitry E, Gasmi M, Raoul JL, Cattan S, Phelip JM, Hammel P, Chauffert B, Michel P, Legoux JL, Rougier P, Bedenne L, Seitz JF, Fédération Francophone de Cancérologie Digestive (2010). Combination 5-fluorouracil, folinic acid and cisplatin (LV5FU2-CDDP) followed by gemcitabine or the reverse sequence in metastatic pancreatic cancer: final results of a randomised strategic phase III trial (FFCD 0301). Gut.

[25] Oettle H, Riess H, Stieler JM, Heil G, Schwaner I, Seraphin J, Görner M, Mölle M, Greten TF, Lakner V, Bischoff S, Sinn M, Dörken B, Pelzer U: Second-Line Oxaliplatin, Folinic Acid, and Fluorouracil Versus Folinic Acid and Fluorouracil Alone for Gemcitabine-Refractory Pancreatic Cancer: Outcomes From the CONKO-003 Trial. J Clin Oncol 2014, in press. 10.1200/JCO.2013.53.6995 24982456

[26] Von Hoff D, LI CP, Wang-Gillam A, Bodoky G, Dean A, Jameson G, Macarulla T, Lee KH, Cunningham D, Blanc JF, Hubner R, Chiu CF, Schwartsmann G, Siveke J, Braiteh F, Moyo V, Belanger B, Dhindsa N, Bayever E, Chen LT, NAPOLI-1 (2014). Randomized phase 3 study of MM-398 (NAL-IRI), with or without 5-Fluorouracil and leucovorin, versus 5-Fluorouracil and leucovorin, in metastatic pancreatic cancer progressed on or following gemcitabine-based therapy. Ann Oncol.

[27] Van Cutsem E, Twelves C, Cassidy J, Allman D, Bajetta E, Boyer M, Bugat R, Findlay M, Frings S, Jahn M, McKendrick J, Osterwalder B, Perez-Manga G, Rosso R, Rougier P, Schmiegel WH, Seitz JF, Thompson P, Vieitez JM, Weitzel C, Harper P, Xeloda Colorectal Cancer Study Group (2001). Oral capecitabine compared with intravenous fluorouracil plus leucovorin in patients with metastatic colorectal cancer: results of a large phase III study. J Clin Oncol.

[28] Hoff PM, Ansari R, Batist G, Cox J, Kocha W, Kuperminc M, Maroun J, Walde D, Weaver C, Harrison E, Burger HU, Osterwalder B, Wong AO, Wong R (2001). Comparison of oral capecitabine versus intravenous fluorouracil plus leucovorin as first-line treatment in 605 patients with metastatic colorectal cancer: results of a randomized phase III study. J Clin Oncol.

[29] de Gramont A, Bosset JF, Milan C, Rougier P, Bouché O, Etienne PL, Morvan F, Louvet C, Guillot T, François E, Bedenne L (1997). Randomized trial comparing monthly low-dose leucovorin and fluorouracil bolus with bimonthly high-dose leucovorin and fluorouracil bolus plus continuous infusion for advanced colorectal cancer: a French intergroup study. J Clin Oncol.

[30] Cassidy J, Clarke S, Díaz-Rubio E, Scheithauer W, Figer A, Wong R, Koski S, Lichinitser M, Yang TS, Rivera F, Couture F, Sirzén F, Saltz L (2008). Randomized phase III study of capecitabine plus oxaliplatin compared with fluorouracil/folinic acid plus oxaliplatin as first-line therapy for metastatic colorectal cancer. J Clin Oncol.

[31] Ducreux M, Bennouna J, Hebbar M, Ychou M, Lledo G, Conroy T, Adenis A, Faroux R, Rebischung C, Bergougnoux L, Kockler L, Douillard JY, GI Group of the French Anti-Cancer Centers (2011). Capecitabine plus oxaliplatin (XELOX) versus 5-fluorouracil/leucovorin plus oxaliplatin (FOLFOX-6) as first-line treatment for metastatic colorectal cancer. Int J Cancer.

[32] Rothenberg ML, Cox JV, Butts C, Navarro M, Bang YJ, Goel R, Gollins S, Siu LL, Laguerre S, Cunningham D (2008). Capecitabine plus oxaliplatin (XELOX) versus 5-fluorouracil/folinic acid plus oxaliplatin (FOLFOX-4) as second-line therapy in metastatic colorectal cancer: a randomized phase III noninferiority study. Ann Oncol.

[33] Aapro MS, Bohlius J, Cameron DA, Dal Lago L, Donnelly JP, Kearney N, Lyman GH, Pettengell R, Tjan-Heijnen VC, Walewski J, Weber DC, Zielinski C, European Organisation for Research and Treatment of Cancer (2011). 2010 update of EORTC guidelines for the use of granulocyte-colony stimulating factor to reduce the incidence of chemotherapy-induced febrile neutropenia in adult patients with lymphoproliferative disorders and solid tumours. Eur J Cancer.

[34] Jamieson NB, Carter CR, McKay CJ, Oien KA (2011). Tissue biomarkers for prognosis in pancreatic ductal adenocarcinoma: a systematic review and meta-analysis. Clin Cancer Res.

[35] Smith RA, Tang J, Tudur-Smith C, Neoptolemos JP, Ghaneh P (2011). Meta-analysis of immunohistochemical prognostic markers in resected pancreatic cancer. Br J Cancer.

[36] Berger SH, Jenh CH, Johnson LF, Berger FG (1985). Thymidylate synthase overproduction and gene amplification in fluorodeoxyuridine-resistant human cells. Mol Pharmacol.

[37] Popat S, Matakidou A, Houlston RS (2004). Thymidylate synthase expression and prognosis in colorectal cancer: a systematic review and meta-analysis. J Clin Oncol.

[38] Infante JR, Matsubayashi H, Sato N, Tonascia J, Klein AP, Riall TA, Yeo C, Iacobuzio-Donahue C, Goggins M (2007). Peritumoral fibroblast SPARC expression and patient outcome with resectable pancreatic adenocarcinoma. J Clin Oncol.

[39] Sinn M, Sinn BV, Striefler JK, Lindner JL, Stieler JM, Lohneis P, Bischoff S, Bläker H, Pelzer U, Bahra M, Dietel M, Dörken B, Oettle H, Riess H, Denkert C (2014). SPARC expression in resected pancreatic cancer patients treated with gemcitabine: results from the CONKO-001 study. Ann Oncol.

[40] Hidalgo M, Plaza C, Illei P, Brachmann C, Heise C, Pierce D, Romano A, Wei X, Lopez-Rios F, Von Hoff D (2014). SPARC analysis in the phase III MPACT trial of nab-paclitaxel (NAB-P) plus gemcitabine (GEM) vs GEM alone for patients with metastatic pancreatic cancer (PC). Ann Oncol.

[41] Bonnetain F, Bonsing B, Conroy T, Dousseau A, Glimelius B, Haustermans K, Lacaine F, Van Laethem JL, Aparicio T, Aust D, Bassi C, Berger V, Chamorey E, Chibaudel B, Dahan L, De Gramont A, Delpero JR, Dervenis C, Ducreux M, Gal J, Gerber E, Ghaneh P, Hammel P, Hendlisz A, Jooste V, Labianca R, Latouche A, Lutz M, Macarulla T, Malka D, Mauer M, Mitry E, Neoptolemos J, Pessaux P, Sauvanet A, Tabernero J, Taieb J, van Tienhoven G, Gourgou-Bourgade S, Bellera C, Mathoulin-Pélissier S, Collette L (2014). Guidelines for time-to-event end-point definitions in trials for pancreatic cancer. Results of the DATECAN initiative (Definition for the Assessment of Time-to-event End-points in CANcer trials). Eur J Cancer.

[42] Louvet C, Labianca R, Hammel P, Lledo G, Zampino MG, André T, Zaniboni A, Ducreux M, Aitini E, Taïeb J, Faroux R, Lepere C, de Gramont A, GERCOR; GISCAD (2005). Gemcitabine in combination with oxaliplatin compared with gemcitabine alone in locally advanced or metastatic pancreatic cancer: results of a GERCOR and GISCAD phase III trial. J Clin Oncol.

[43] Safran H, Perez K, Charpentier K, Clark Austin T, Mantripragada KC, Bishop KD, Lombardo A, Houlihan L, Mitchell K, Rosati K, Martel D, Shaw L (2014). Nab-paclitaxel (nab-P) combined with FOLFOX for advanced pancreatic cancer: A phase I study. J Clin Oncol.

[44] Trouilloud I, Dupont-Gossard AC, Artru P, Lecomte T, Gauthier M, Aparicio T, Thirot-Bidault A, Malka D, Lobry C, Asnacios A, Lacombe S, Fein F, Fanica D, Dubreuil O, Marthey L, Zaanan A, Bonnetain F, Taïeb J (2012). FOLFIRI.3 (CPT-11 plus folinic acid plus 5-FU) alternating with gemcitabine or gemcitabine (G) alone in patients (pts) with previously untreated metastatic pancreatic adenocarcinoma (MPA): Results of the randomized multicenter AGEO phase II trial FIRGEM. J Clin Oncol.

